# A Case in Practice

**Published:** 1880-12-20

**Authors:** J. Hendree

**Affiliations:** Alabama


### A CASE IN PRACTICE.

BY J. HENDREE, M.D., OF ALA.

A few weeks since I was called to see a negro child one year old, which had been affected with spasms, not very violent, during three days. As I could find no indications of malarial fever I felt satisfied that some accident was the cause of the symptoms. Upon questioning the mother very closely she recalled that a week previous to my visit the child laid upon a blanket in the garden, grasped at a large rooster passing near, and the fowl struck its spur into the scalp. There was considerable outcry at the time, but the wound seemed to have healed and the mother had almost forgotten the circumstance. I then examined the scalp and found a tender spot and minute fistulous openings over the lambdoid suture on the right side, quite near the fontanelle. Enlarging this somewhat with a lancet gave egress to a considerable quantity of pus and bloody serum, which was increased by placing that part of the head in a dependent position. I directed this position to be kept as far as practicable. Relief of spasmodic condition was only temporay, and death insued in forty-eight hours. This was really a case of death from the spur of a cock.

